# Nutritional insights into pulmonary fibrosis: a comprehensive review on the impact of vitamins

**DOI:** 10.3389/fnut.2025.1525408

**Published:** 2025-04-11

**Authors:** Yaqian Qu, Youliang Zhao

**Affiliations:** ^1^Henan University of Traditional Chinese Medicine, Zhengzhou, Henan, China; ^2^Department of Occupational and Environmental Health, College of Public Health, Zhengzhou University, Zhengzhou, Henan, China

**Keywords:** pulmonary fibrosis, vitamin, dietary nutrition, EMT, vitamin deficiency

## Abstract

Pulmonary fibrosis is a fatal interstitial disease characterized by diffuse alveolitis, abnormal fibroblast proliferation, and extracellular matrix (ECM) accumulation, resulting in structural lung destruction and impaired lung function. Numerous studies have demonstrated that vitamins appear to play a crucial role in regulating inflammatory responses, cell differentiation, redox homeostasis, and collagen synthesis. Beyond their conventional nutritional functions, specific vitamins have recently been found to modulate various biological processes involved in pulmonary fibrosis. This study aims to provide a comprehensive overview of the current understanding regarding the impact of vitamins on pulmonary fibrotic disease.

## 1 Introduction

Pulmonary fibrosis is a chronic pulmonary disease characterized by the progressive accumulation of extracellular matrix (ECM) proteins, resulting in the formation of scar tissue and thickening of connective tissue, and the pathological process is often accompanied by persistent tissue inflammation (1). This refractory pulmonary disorder is characterized by progressive and irreversible destruction of lung structure caused by scar, ultimately leading to lung dysfunction, gas exchange obstruction, and respiratory failure (1). Currently, there are no effective therapeutic interventions available for pulmonary fibrosis apart from lung transplantation.

In recent years, emerging research of healthy diet and nutrition interventions in preventing or alleviating various chronic diseases leading to substantial pharmaceutical investments. Numerous studies have been devoted to demonstrating the intricate relationship between nutrients and diverse chronic diseases. For instance, dietary interventions play a pivotal role in effectively managing metabolic disorders such as diabetes mellitus (2); and dietary modifications hold substantial value in ameliorating renal failure (3); additionally, a well-established association exists between malnutrition and immune system dysregulation (4); furthermore, the association between nutrition and cancer has also garnered considerable interest (5). New evidence suggests that there may be a substantial link between dietary nutrient intake and lung health, as supported by epidemiological studies demonstrating that the consumption of antioxidant-rich foods can mitigate the risk of developing chronic lung disease (6–8). Additionally, the incorporation of fresh fruits and vegetables into one's diet appears to confer protection against lung disease.

Multivitamins, widely used as nutritional supplements, have garnered significant attention in the scientific community due to their potential for preventive or ameliorative effects on various chronic diseases. Many chronic diseases share common pathogenesis that can be modulated by vitamins, such as folic acid regulates DNA methylation (9), vitamin D governs bone mineral density (10), and both vitamin A and vitamin D regulate cell proliferation and differentiation (11, 12). In pulmonary fibrosis, a case-control study in Japan investigated the association between vegetable, fruit, grain, and fiber intake and the risk of idiopathic pulmonary fibrosis (IPF), it was observed that there is a beneficial association between higher fruit intake and IPF, although larger studies with more details information are warranted to confirm it (13). Moreover, numerous studies have found an association between hypovitaminosis and pulmonary fibrosis and attempted to explore the content characteristics of specific micronutrients (including vitamins) and the possibility of alleviation or treatment for pulmonary fibrosis (14, 15).

Despite numerous studies have investigated the roles of different vitamins in various pulmonary diseases over the past decades, there has been a limited coverage or even neglect of their specific protective activities in relation to pulmonary fibrosis within otherwise comprehensive reviews. This review aims to elucidate the pathophysiology of pulmonary fibrosis and provide a summary of the involvement of vitamins in the development and progression of this condition, thereby offering valuable insights into potential vitamin targets for pulmonary fibrosis.

## 2 Pathogenesis of pulmonary fibrosis

**Figure 1:**
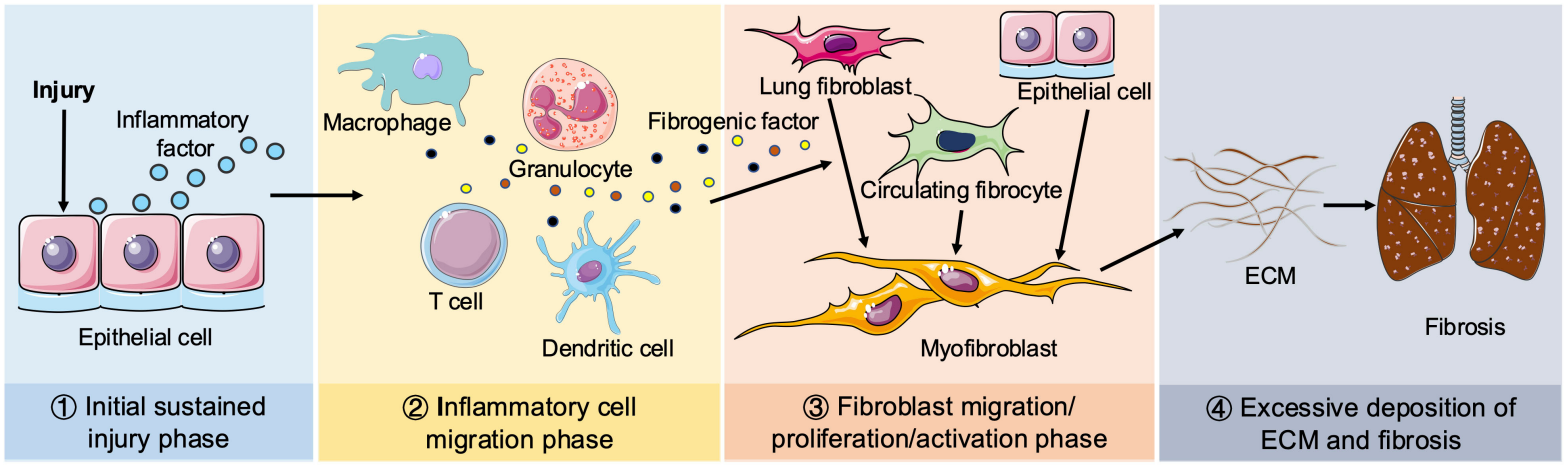
Schematic diagram of pulmonary fibrosis. Four phases of pathological process of pulmonary fibrosis: ① Initial sustained injury phase. ② Inflammatory cell migration phase. ③ Fibroblast migration/proliferation/activation phase. ④ Excessive deposition of ECM and fibrosis phage. When lungs are continuously stimulated by the external environment, epithelial cells release inflammatory mediators. Next, various inflammatory cells (including macrophages, granulocytes, T cells and dendritic cells, etc.) are recruited to the damaged tissue to remove foreign substances and secrete multiple fibrogenic factors, including TGF-β, IL-β1, IL-13, TNF-α. Circulating fibroblasts and interstitial fibroblasts subsequently proliferate and differentiate into myofibroblasts. Epithelial cells can also be transformed into myofibroblasts by EMT. Sustained injury causes the disorder of myofibroblasts secreting ECM to promote the wound repair process, resulting in excessive deposition of ECM and ultimate pulmonary fibrosis.

Tissue repair following injury is an inherent biological mechanism that restores tissue integrity through the organized replacement of damaged or deceased cells (16). However, dysregulation of this reparative process leads to excessive proliferation of myofibroblasts and aberrant secretion of ECM, resulting in permanent fibrous scarring at the site of tissue injury. Consequently, pulmonary fibrosis represents an uncontrolled hyper-reparative response to tissue wounds. The pathological process of pulmonary fibrosis can be roughly divided into four phases, as illustrated in Figure 1: the initial sustained injury phase, the inflammatory cell migration phase, the fibroblast migration/proliferation/activation phase and finally, the excessive deposition of ECM and fibrosis phase. The immediate pathological manifestations of pulmonary fibrosis involve injury to alveolar epithelial cells accompanied by secretion of inflammatory factors (17). There is efficient recruitment of inflammatory cells to the site of injury for clearance, concomitant with production of various cytokines and chemokines such as TGF-β, IL-1β, IL-13, TNF-α, etc., which stimulate proliferation and recruitment of lung fibroblasts. Myofibroblasts predominantly arise from three distinct pathways: (1) activation of interstitial fibroblasts; (2) epithelial mesenchymal transition (EMT); and (3) migration of bone marrow fibroblasts. Myofibroblasts secrete ECM, thereby facilitating wound contraction. While an appropriate ECM quantity aids in tissue damage repair, persistent injury leads to dysregulated excessive ECM deposition, resulting in the replacement of normal lung tissue with fibrous scars (18).

## 3 Discovery of vitamins

Vitamins are a group of organic substances that play an essential role as micronutrients in cellular metabolism and the overall maintenance of organismal health. In 1906, Frederick Hopkins first proposed the importance of vitamins through experiments. He demonstrated that rats fed only proteins, fats, carbohydrates, and minerals failed to grow properly, but exhibited improved growth when supplemented with a small amount of milk. This led him to suggest the existence of “accessory factors” in food that were crucial for life processes, in addition to the known major nutrients. These “accessory factors” were later identified as vitamins. In 1912, Casimir Funk coined the term “vitamine” (meaning “vital amine”) for these substances. When scientists subsequently discovered that not all vitamins contained amine structures, the final “e” was dropped, resulting in the modern term “vitamin” (19, 20). Vitamins can be classified into water-soluble types, such as vitamin B and C, and fat-soluble types, including vitamin A, D, E, and K. Each vitamin has distinct optimal food sources, and their deficiencies can cause a variety of diseases (Table 1).

**Table 1:** The main information about vitamins mentioned in this review.

**Classification**	**Vitamins**	**Main food sources**	Recommend daily dose^*^	**Upper daily intake**
Water-soluble vitamins	Vitamin B1 (Thiamine)	whole grains; legumes; nuts; lean meats; yeast; fortified cereals	M: 1.2 mg F: 1.1 mg	ND
	Vitamin B2 (Riboflavin)	milk; dairy products; eggs; lean meat; green leafy vegetables; fortified cereals	M: 1.3 mg F: 1.1 mg	ND
	Vitamin B3 (Niacin)	Poultry; egg; fish; peas; mushroom; asparagus; peanut	M: 16 mg F: 14 mg	ND
	Vitamin B5 (Pantothenic acid)	meats; whole grains; legumes; eggs; milk	5 mg	ND
	Vitamin B6 (Pyridoxine)	Fish; poultry; legumes; potatoes; bananas; fortified cereals	Adults (19–50 years old): 1.3 mg Men over 50 years old: 1.7 mg Women over 50 years old: 1.5 mg	100 mg
	Vitamin B7 (Biotin)	Egg yolks; liver; yeast; nuts, legumes; whole grains	30 μg	ND
	Vitamin B9 (Folic acid)	Liver; kidney; egg; beans; yeast; nut; green vegetable; fruit	400 μg	ND
	Vitamin B12 (Cobalamin)	Meats; fish; dairy products; eggs; fortified cereals	2.4 μg	ND
	Vitamin C (Ascorbic acid)	Fruits; green vegetable; tomato; potato	M: 90 mg F: 75 mg	2,000 mg
Fat-soluble vitamins	Vitamin A (Retinol)	Carrots; green vegetable; yelk; liver; milk; coriander; grape	M: 1.2 mg F: 1.3 mg	3,000 mcg
	Vitamin D (Calciferol)	Cod liver oil; dairy products; egg	15 μg	100 mcg
	Vitamin E (Tocopherol)	Vegetable oil; germ of grain; beans; gingili; peanut; vegetable; milk	15 mg	1,000 mg
	Vitamin K (Phylloquinone)	green vegetable; fruits; liver; meat; milk; yelk	M: 120 μg F: 90 μg	ND

^*^Adults in non-special period; M, Male; F, Female; ND, Not determinable.

## 4 Vitamins and pulmonary fibrosis

### 4.1 Vitamin A

Vitamin A, derived from carotenoid pigments in nature (known as provitamin A), belongs to the class of fat-soluble micronutrients and undergoes two consecutive oxidation reactions to convert into its primary biologically active derivatives, retinal and retinoic acid (RA). Vitamin A serves multiple crucial functions within the human body, encompassing cell proliferation and differentiation, vision, immunity, and embryological development (21–25). Moreover, it has been proved to be intricately associated with numerous respiratory defense mechanisms and assumes a pivotal role in upholding the structural integrity of respiratory mucosa (26, 27). Vitamin A is also necessary for maintaining alveolar structure and tissue regeneration, and trophic vitamin A deficiency (VAD) leads to detrimental histological alterations in lung parenchyma which predispose to severe tissue dysfunction and respiratory diseases, even increasing the incidence and morbidity of patients, suggesting that vitamin A plays an important role in adult lungs (28–30).

The excessive deposition of ECM is widely recognized as the primary characteristic of pulmonary interstitial fibrosis. Pathological changes in lung tissue induced by VAD encompass alterations in both the content and distribution of ECM proteins (31–33). Retinoid signaling directly or indirectly modulates gene promoters, thereby participating in the regulation of ECM protein expression and influencing the expression of ECM receptor proteins on cell membranes (34). Guillermo et al. observed elevated levels of collagen I and collagen IV in vitamin A-deficient rat lungs, accompanied by a nearly twofold increase in alveolar basement membrane thickness and ectopic deposition of collagen I protein, while treatment with retinoic acid reversed these alterations (35). The mechanism underlying the alteration of ECM by VAD remains elusive, but there is some evidence. Dysregulation of transforming growth factor-β1 (TGF-β1)/Smad3 signaling pathway is considered to play a central role and is associated with bleomycin-induced pulmonary fibrosis (36). Additionally, Chiharu et al. reported all-trans retinoic acid (ATRA) inhibits both proliferation and transdifferentiation of lung fibroblasts through downregulating TGF-β expression and suppressing the IL-6/IL-6R system (IL-6 stimulates lung fibroblast proliferation in a dose-dependent manner), thereby preventing radiation- and bleomycin-induced pulmonary fibrosis in mice (37). Inhibition of IL-6 and TGF-β production by ATRA was achieved by blocking the PKC-δ/NF-κB and p38MAPK/NF-κB pathways (38) and shifting the regulatory T/T helper 17 ratio and reducing the secretion of IL-17A (39). Vitamin A also affects the EMT process of lung epithelial cells, the effect of retinoic acid in inhibiting this process has been demonstrated (40, 41). In lungs of VAD rats, there is an increased level of N-cadherin protein accompanying decreased levels of E-cadherin and β-catenin (42). Supplementation with ATRA improves alveolar septal defect at the tissue level and promotes alveolar epithelial recovery (43). All these findings collectively suggest a potential association between vitamin A and EMT process in lung fibrosis.

Patients with lung fibrosis exhibit an elevated oxidative burden and heightened levels of reactive oxygen species (ROS) generated by inflammatory cells in lungs (44, 45). ROS have been demonstrated to activate transcription factors, such as AP-1, thereby inducing the synthesis of fibrogenic factor TGF-β, various ECM proteins, as well as type I and type IV collagens (46–48). Early research on the antioxidant properties of vitamin A came from Monaghan & Schmitt, who found that dissolving fresh vitamin A in linoleic acid hindered the absorption of oxygen by linoleic acid, and this effect faded away as vitamin A was oxidized (49). Burton and Ingold proposed that beta-carotene (which is converted into vitamin A in body) and other carotenoids may play an important role in protecting tissue lipids against peroxidation *in vivo* (50). Both carotenoids with provitamin A activity and non-vitamin A carotenoids exert antioxidant functions in lipid phases by quenching free radicals or O_2_ (51). Vitamin A and its metabolites have been frequently recognized as notable antioxidants in various tissues, including the lung, liver, and heart (52). Levels of retinol, one of the forms of vitamin A, are elevated in the bronchoalveolar lavage fluid of patients with IPF, and this increase may be part of an adaptive response to oxidative stress (14). Another study proved that VAD can disrupt the balance between ROS production and antioxidant defense mechanisms in lungs (31, 53). The above information suggests a potential association between vitamin A and pulmonary fibrosis through the redox pathway. However, experimental confirmation is required to determine whether vitamin A supplementation can effectively ameliorate pulmonary fibrosis via modulation of the redox pathway. It is worth noting that long-term excessive intake of vitamin A may disrupt the immune balance of the body (24, 54, 55). In the occurrence and development of pulmonary fibrosis, the imbalance of the immune system is an important factor (56). It is speculated from this that excessive intake of vitamin A may alter the functions and activities of immune cells, which indirectly aggravates pulmonary fibrosis.

### 4.2 Vitamin B

Vitamin B refers to a group of eight water-soluble vitamins that play crucial roles in cellular metabolism. Except for vitamin B3 (niacin), which can be partially synthesized in the human body from tryptophan, the other vitamins in this group cannot be synthesized by the body and must be obtained through dietary sources. All eight components: vitamin B1 (thiamine), vitamin B2 (riboflavin), vitamin B3 (niacin or niacinamide), vitamin B5 (pantothenic acid), vitamin B6 (pyridoxine, pyridoxal, or pyridoxamine, or pyridoxine hydrochloride), vitamin B7 (biotin), vitamin B9 (folic acid) and vitamin B12. Each individual component of vitamin B possesses its own distinct structure and exerts unique physiological functions within the human body.

#### 4.2.1 Niacin

Research on vitamin B and pulmonary fibrosis has mainly focused on niacin at the end of the last century. Niacin serves as a precursor for nicotinamide adenine dinucleotide (NAD, a substrate for poly (ADPribose) polymerase) and nicotinamide adenine dinucleotide phosphate (NADP) and participates in DNA repair. In 1989, Wang et al. reported its potential anti-fibrosis function by attenuating bleomycin-induced thickened alveolar septa, accumulation of inflammatory cells and foci of fibrotic consolidation (57). DNA damage is one of the mechanisms of bleomycin-induced lung injury. One plausible mechanism involves the conversion of niacin into NAD in animals, thereby creating an environment with ample NAD supply that enhances the DNA repair capacity of poly (ADP-ribose) polymerase (a key enzyme regulating cell proliferation and repair in injured lungs) upon stimulation by bleomycin (57, 58). Calcium can activate phospholipase A2 (PLA2) to exacerbate pulmonary inflammatory events. Niacin, on the other hand, may inhibit bleomycin-induced pulmonary fibrosis by suppressing PLA2 activity through the prevention of calcium influx (59). Despite reports of fatty liver disease and growth inhibition in rats with long-term niacin administration, this compound is generally considered non-toxic because it is easily metabolized and excreted (60). In fact, niacin has been clinically utilized at supraphysiological doses as a vasodilator and cholesterol-lowering drug for lipid management (61, 62). Subsequently, numerous studies have analyzed the protective effect of niacin in combination with taurine on pulmonary fibrosis, and the possible mechanisms are as follows: 1) Niacin and taurine treatment suppressed bleomycin-induced transcriptional nuclear factor-NF-κB by preserving IκBα (63) to inhibit the increased levels of inflammatory cell-derived fibrocytokines such as IL-1α, IL-6, TNF-α and TGF-β (64). 2) Niacin and taurine treatment suppressed the expression of procollagen I and procollagen III at a transcription level (65), this may be related to the reduction of lung prolyl hydroxylase activity since the increased activity of this enzyme is positively correlated with and preferentially expressed in the accumulation of collagen in lungs (66). Additionally, niacin and taurine exhibited inhibition of nitric oxide production induced by iNOS in lungs (67).

#### 4.2.2 Folic acid

Folic acid donates a carbon unit during DNA biosynthesis and is important for the regulation of gene expression, transcription, chromatin structure, genomic repair and genomic stability (68). No previous studies have considered the possible relationship between folic acid and pulmonary fibrosis even though DNA damage is involved in this disease. In recent years, studies have revealed an upregulation of folate receptor-β expression in alveolar macrophages of patients with IPF compared to normal alveolar macrophages, which mediates the unidirectional transport of folates into cells (69). Some high-risk factors for IPF disease, such as smoking, can lead to abnormal epithelial barrier and impede folate absorption in patients (40, 70). Furthermore, folate is required for the proliferation of fibroblasts and activated macrophages during pulmonary fibrosis. These facts seem to establish a link between folic acid and pulmonary fibrosis. Although inhibition of fibroblast proliferation is a way to reduce pulmonary fibrosis and it seems necessary to block folic acid absorption, cortisone-based drugs that block folic acid absorption have yielded unsatisfactory outcomes. Conversely, considering that oxidative stress plays a crucial role in the development of pulmonary fibrosis, folic acid as a highly reductive vitamin may have a favorable impact on its prevention or treatment. Our study showed that folic acid supplementation can promote the expression of SLC25A32 and MTHFD2, which are key enzymes in mitochondrial folate metabolism. It can enhance the mitochondrial folate metabolic pathway and reduce the production of mitochondrial ROS, thereby inhibiting the progression of pulmonary fibrosis (71). Another strategy worth considering is the conjugation of folic acid to the drug, enabling cellular entry and exertion of its effect through binding with folate receptors on activated macrophages (72). Of course, further experimental validation is necessary to elucidate the role of folic acid in pulmonary fibrosis.

B-group vitamins are water-soluble, the excessive amount is generally excreted out of the body through urine. This means that they are generally safe even at doses higher than the Recommended Dietary Allowance (RDA). Only three of the B-vitamins have been set an upper limit for daily intake, while the remaining ones are considered safe regardless of the intake dosage (73, 74). The first one is folic acid. Its Recommended Dietary Allowance (RDA) per day is typically between 200 and 400 micrograms, and the upper limit of its intake is generally set at 1,000 micrograms per day. This is simply because an increase in folic acid intake may mask the symptoms of vitamin B12 deficiency, allowing permanent damage related to the latter vitamin to accumulate unknowingly (75). It should also be noted that there is evidence suggesting that the intake of high doses of folic acid, and the resulting increase in the levels of unmetabolized folic acid, may have potential adverse effects on normal folic acid metabolism and immune function. However, up to now, there is no consensus on the blood concentration of folic acid that might cause harm (76). The second one is niacin, and its upper limit of intake is set at 35 milligrams (in the United States/Canada). This is simply because when the dosage exceeds 100 milligrams, it will cause temporary skin flushing. However, after long-term intake of a dosage of 1 gram or more, symptoms such as nausea, vomiting, and diarrhea have been observed, and in extremely rare cases, liver damage may also occur (77). The last B-vitamin with a set upper intake limit is vitamin B6. In the United States, its upper limit is set at 100 mg per day (roughly 75 times the Recommended Dietary Allowance), which is based on case reports showing that reversible sensory neuropathy occurs after long-term use of doses exceeding 1,000 mg (78). Currently, there are relatively few studies on the direct impact of an excess of B-group vitamins on pulmonary fibrosis. Overall, there is no conclusive evidence indicating that an excess of B-group vitamins can directly lead to pulmonary fibrosis or have a definite and severe adverse effect on it.

### 4.3 Vitamin C

Vitamin C, scientifically referred to as ascorbic acid, is an indispensable micronutrient and a coenzyme that potentiates the activity of diverse enzymes, contributing to the immune response and the development of nervous system in humans (79, 80). Severe deficiency of vitamin C can lead to the occurrence of many serious diseases. Due to the limited ability of organisms to preserve this essential nutrient, continuous intake is necessary to prevent deficiencies. Study has shown that silicosis patients exhibit reduced serum levels of vitamin C compared to individuals not exposed to silica (81). In studies investigating the efficacy of vitamin C treatment for pulmonary fibrosis, Vildan et al. demonstrated that vitamin C did not yield significant improvements in IPF symptoms in a rat model (82). However, other studies showed that administration of vitamins C and E both alleviate fibrotic damage in lung tissue, and with a more pronounced effect observed when these two vitamins are combined in rat models (83). Furthermore, the administration of vitamin C has been shown to protect against collagen I and α-SMA deposition (84). In general, the anti-pulmonary fibrosis effect of vitamin C is primarily manifested in three aspects: anti-oxidation, anti-cell death and anti-inflammation.

#### 4.3.1 Anti-oxidation function

Oxidative stress caused by high concentration of oxygen in the lungs can lead to the dysfunction of alveolar epithelial cells, thereby causing certain damage to the lung tissue. Among small antioxidants, glutathione has garnered significant attention, however, as one of the main cellular antioxidants, vitamin C surpasses glutathione by directly detoxifying ROS. Vitamin C acts as a natural antioxidant by readily donating electrons to safeguard crucial biomolecules (proteins, lipids, carbohydrates, and nucleic acids) against oxidative damage arising from cellular metabolism (85). Furthermore, it reduces the content of ROS and reactive nitrogen species (RNS) by giving electrons to them and preventing the oxidation of other compounds (86). Vitamin C also regenerates other antioxidant molecules such as glutathione (GSH), beta-carotene, urate, alpha-tocopherol and membrane antioxidants glutathione and vitamin E (87–89). Despite being highly hydrophilic in nature, vitamin C effectively inhibits lipid oxidation by reducing tocopherol free radicals and thus maintaining optimal levels of vitamin E, a major fat-soluble antioxidant (90).

While these molecular mechanisms highlight vitamin C's antioxidative potential in pulmonary systems, emerging clinical evidence further explores its therapeutic implications for fibrotic lung diseases. Vitamin C supplementation for a duration of 12 weeks demonstrates significant reduction in serum protein carbonyl levels, which serves as a reliable marker for evaluating the antioxidant status in patients with IPF (91). However, whether vitamin C can definitively alleviate pulmonary fibrosis by lowering serum protein carbonyl levels still lacks concrete evidence. Additionally, it actively participates in collagen biosynthesis, acts as a cofactor for hydroxylases, and regulates gene expression (92, 93). In cystic fibrosis, vitamin C serves as a crucial source of glutathione and aids in maintaining the delicate balance between oxidative stress and antioxidant defense mechanisms within the lungs (94). Moreover, through its modulation of Nrf2/Nox4 redox equilibrium and TGF-β1/Smad3 signaling pathways, vitamin C exerts a protective effect against PQ-induced pulmonary fibrosis (95).

#### 4.3.2 Anti-cell death function

The potential role of vitamin C in cell death has long been proposed (96), and significant advancements have been made across various types of cell death. Herein, we aim to concisely summarize these findings and elucidate the regulatory mechanisms by which vitamin C modulates cell death in pulmonary fibrosis.

##### 4.3.2.1 Anti-apoptosis function

The prevailing perspective suggests that the development of pulmonary fibrosis necessitates repetitive damage to epithelial cells and subsequent apoptosis, with documented instances of alveolar epithelial cell (AEC) apoptosis in lung fibrotic lesions (97). Apoptosis of alveolar type II epithelial cells was also observed in bleomycin- or thoracic irradiation-treated mice (98, 99). Similarly, a study demonstrated that pro-apoptotic markers (p53, p21, Bax, and caspase-3) stained positive in type II AECs from IPF subjects, while anti-apoptotic markers (Bcl-2) exhibited reduced expression compared to control subjects (100). Moreover, the increased apoptosis of type II AECs in IPF patients can directly cause alveolar collapse, thus accelerating the progression of pulmonary interstitial fibrosis (101). In animal models, it has been reported that the activation of apoptosis in type II AEC promotes a fibrotic phenotype, for example, apoptotic cells may release cytokines and growth factors that recruit and activate fibroblasts, prompting them to secrete ECM, whereas inhibiting apoptosis helps to alleviate pulmonary fibrosis (102–104). Although the impact of vitamin C on type II AEC in the pathogenesis of pulmonary fibrosis remains limited, evidence suggests that vitamin C can enhance epithelial barrier function through diverse mechanisms. The administration of vitamin C can significantly alleviate the pro-inflammatory response and the aggregation of polymorphonuclear neutrophils in the lungs of infected mice, and effectively restore the function of the lung epithelial barrier that has been damaged by severe infections (105). Another, in an animal experiment on N-Nitrosodimethylamine (NDMA)-induced pulmonary fibrosis, ascorbic acid was observed to significantly reduce P53, caspase-3, Bax (which inhibits the anti-apoptotic effect of Bcl-2) and Bax/Bcl-2 ratio in lung tissue, while increasing Bcl-2 and mdm2 levels, thereby exerting anti-apoptotic properties (84). Heleen M et al. have also demonstrated the enhancement of vitamin C on the anti-apoptotic ability of Bcl-2 and its inhibitory effect on the expression of Bax protein, thereby impeding mitochondrial release of cytochrome C to the cytoplasm, ultimately reducing caspase-3 activity and suppressing cell apoptosis (106). However, further investigations are warranted to elucidate the mechanism by which vitamin C counteracts apoptosis in alveolar type II epithelial cells as well as its impact on apoptosis in other lung cell types.

##### 4.3.2.2 Anti-ferroptosis

Ferroptosis, a newly discovered type of programmed cell death, occurs in an iron-dependent manner. As an indispensable trace element in the human body, the imbalance of iron homeostasis may lead to a variety of physiological disorders, including lung fibrotic disease (107). Histological samples from patients with IPF showed accumulation of extracellular iron and hemosiderin within macrophages, and this phenomenon is closely associated with reduced total lung capacity and lung compliance (107–110). In addition, iron overload was found to significantly increase lung inflammation and oxidative stress in mice, which are also crucial mechanisms underlying pulmonary fibrosis (111). *In vitro*, high iron level have been shown to directly stimulate the proliferation of human lung fibroblasts and enhance the secretion of ECM and pro-inflammatory factors (107). These findings suggest that there may be a close underlying association between iron overload and the development of pulmonary fibrosis. Ferroptosis will be triggered when iron overload surpasses the self-regulatory capacity of cells. Furthermore, ferroptosis can be inhibited by glutathione (a water-soluble antioxidant) or α-tocopherol (a lipid-soluble antioxidant), indicating that ferroptosis is not solely dependent on iron but is also closely associated with oxidative stress (112). Ferrostatin-1 and Liproxstatin-1, specific inhibitors of ferroptosis, work by inhibiting the accumulation of lipid hydroperoxides, which emphasizes the key role of lipid peroxidation in ferroptosis (113). Studies have demonstrated that enhancement of ROS and lipid peroxidation is one of the culprits in the elevation of α-SMA and COL1A1, indicative of myofibroblast formation. The effect of vitamin C on ferroptosis is concentration-dependent. At physiological concentrations, vitamin C behaves as a suppressant of ferroptosis induced by erastin or RSL3 (114). In contrast, at high concentrations, the cytotoxic effects of vitamin C outweigh its protective properties. Specific ferroptosis inhibitors were not effective for tumor cells (HT-1080/MCF-7) treated with ascorbic acid at pharmacologic concentration (114). However, the presence of pyruvate can effectively improve the inhibitory function of high concentration of ascorbic acid on ferroptosis (114). Ferroptosis agonists directly or indirectly affect glutathione peroxidase (GPXs) through various pathways, leading to a reduction in antioxidant capacity, accumulation of ROS, and ultimately oxidative cell death (115, 116). Based on this, it can be inferred that the anti-ferroptosis function of vitamin C at physiological concentrations may stem from its antioxidant property.

#### 4.3.3 Anti-inflammation

The development of lung fibrosis is mostly the result of the development of prior chronic lung inflammation. Alveolar macrophages serve as the first cells to encounter external pathogens and irritants, initiating and subsequently resolving the lung immune response. In response to lung injury, macrophages activated by LPS and IFNγ undergo a transition to the pro-inflammatory M1 phenotype and begin to secrete pro-inflammatory cytokines and chemokines, resulting in enhanced chemotaxis of monocytes and neutrophils (117, 118). Simultaneously, neutrophils also release a plethora of inflammatory mediators. During chronic inflammation, myofibroblasts evade apoptosis and contribute to aberrant wound healing processes characterized by excessive extracellular matrix (ECM) production, ultimately culminating in pulmonary fibrosis. Therefore, reducing lung inflammation seems to be a feasible way to prevent lung tissue fibrosis and has been substantiated through scientific investigations. For example, Cabozantinib ameliorates lipopolysaccharide-induced lung inflammation in mice and inhibited bleomycin-induced early pulmonary fibrosis (119); Thymosin β4 suppresses LPS-induced murine lung fibrosis by alleviating inflammation (120).

The elasticity of the lung tissue in IPF patients is irreversibly reduced, which seriously affects the respiratory function (121). Masha et al. demonstrated although vitamin C, D, and E supplementation had no effect on exhaled parameters, it successfully improved inhaled parameters, especially total lung capacity (TLC) and residual volume (RV), which are the most critical indicators of lung function (122, 123). This implies that vitamins C, D, and E have the potential to enhance maximal oxygen uptake and inspiratory muscle strength in IPF patients. Previous studies have also demonstrated that vitamin C exerts anti-inflammatory effects through modulation of both adaptive and innate immunity (124, 125). Neutrophils are short-lived cells that continuously produced by the bone marrow and delivered to blood, and rapidly migrate into the inflamed tissue during an innate inflammation. Lymphocytes are long-lived cells that infiltrate tissue during an acquired inflammation. Studies have shown that vitamin C treatment reduces leukocyte recruitment in mice with pulmonary fibrosis by paraquat (126), which may indicate a direct relationship between vitamin C and immune cells. Nevertheless, data also showed that neutrophils accumulate a large amount of vitamin C, similarly, lymphocytes also accumulate vitamin C to regulate cell proliferation and the function of B cells and T cells (79), which help protect cells from oxidative stress damage during inflammation. IL-17, which has a variable inflammatory effect that promotes the migration of inflammatory cells to inflammatory tissues, levels of IL-17 were found to decrease in lung homogenates of fibrotic mice treated with vitamin C (126). Therefore, the therapeutic efficacy of vitamin C in treating pulmonary fibrosis appears to be attributed, at least in part, to the attenuation of pro-inflammatory factors secreted by resident or migratory cells.

It is noteworthy that in certain cases, vitamin C may reduce the body's immune response. High doses of vitamin C can inhibit the proliferation of natural killer cells as well as T and B lymphocytes responsible for the secretion of interleukin-2 (IL-2). Additionally, vitamin C can block the activation of T lymphocytes, which are forced into an immature state after stimulation by dendritic cells (127). However, there has been no research on whether excessive intake of vitamin C has an adverse impact on patients with pulmonary fibrosis.

### 4.4 Vitamin D

Vitamin D is widely distributed throughout the body, not only in the liver but also in various tissues. Numerous hydroxylases have been discovered in these tissues, facilitating the conversion of vitamin D to 1,25-dihydroxyvitamin D. This active form is believed to exert its primary function by binding to the ligand-dependent transcription factor known as the vitamin D receptor (VDR) (128–131). Vitamin D deficiency is a prominent characteristic of physiological aging, with its concentration declining as individuals age (132). Emerging research indicates that vitamin D insufficiency exacerbates cellular aging across various cell types. Supplementation with vitamin D has conventionally been postulated to mitigate the aging process in bone and muscle cells, thereby preserving or even enhancing their overall health (133–135). Vitamin D deficiency is strongly associated with various lung diseases, including asthma and chronic obstructive pulmonary disease (COPD). Low levels of 25-hydroxyvitamin D may contribute to the development or exacerbation of these conditions (136). Furthermore, there is evidence supporting a link between vitamin D deficiency and pulmonary fibrosis. For instance, Zhou et al. found that lung Sirt1 and serum vitamin D levels declined during physiological aging, thus activating the TIME signaling pathway, and promoting senescence-associated pulmonary fibrosis (137). Li et al. discovered that vitamin D deficiency aggravated bleomycin-induced pulmonary fibrosis (138). Notably, in this study, vitamin D appears to influence the development of pulmonary fibrosis through multiple biological pathways: vitamin D deficiency not only exacerbates the lung inflammation caused by bleomycin (similar to the effect observed in the case of vitamin C deficiency), but also increases the likelihood of the occurrence of EMT in the lungs, and these are all crucial steps in the pathogenesis of pulmonary fibrosis. Additionally, it exacerbates pulmonary fibrosis by activating the TGF-β/smad3 pathway (138). Furthermore, several small-scale prospective studies have consistently demonstrated a prevalent insufficiency or deficiency of plasma vitamin D levels in patients with IPF, which is strongly correlated with acute exacerbations of the disease (139, 140). However, it should be noted that there remains a dearth of large-scale randomized controlled trials investigating the potential role of vitamin D supplementation in improving IPF outcomes.

Conversely, vitamin D supplementation has demonstrated the potential to enhance pulmonary function and impede fibrosis progression. Studies have indicated that the inhibitory effect of vitamin D on pulmonary fibrosis manifests across various stages of disease advancement. (1) In the early stage, vitamin D plays an anticoagulant role. Epithelial cells undergo damage and subsequently release inflammatory mediators that activate the anti-fibrinolytic coagulation cascade. Furthermore, studies have revealed that the overactivation of the clotting cascade permeates the entire process of pulmonary fibrosis (141, 142). Coagulation factors are mainly mediated by protease activating receptors (PARs) (141). PARs mediate tissue factor TF, which then works with activating factor VIIa (FVIIa) to form the TF/FVIIa complex (the main promoter of the clotting cascade), and this pathway can be blocked by TFPI, a protease inhibitor (143, 144). Studies have shown that vitamin D may interfere with the above clotting response in two ways: Vitamin D inhibits TF expression through TNF-α (145–147); Vitamin D is potentially involved in the regulation of TFPI expression, as several studies have demonstrated a positive correlation between their levels (146). (2) Excessive inflammatory mediators trigger the infiltration of inflammatory cells, which then release a multitude of cytokines, such as transforming growth factors and fibroblast growth factors. These released factors, in turn, promote both inflammation and fibrosis (148–150). Previous studies have shown that inflammatory mediators play an important role in the occurrence and development of pulmonary fibrosis. Vitamin D has been shown to reduce serum levels of inflammatory cytokines, including IL-3 (151), IL-17 (151, 152), IL-1, IL-6, IL-8, and TNF-α (153), and may directly act on CD4^+^ T cells to promote the secretion of the anti-inflammatory cytokine IL-10 by T regulatory cells (Tregs), controlling the further expansion of the inflammatory response (151, 152, 154, 155). (3) The activation of the EMT process by TGF-β plays a pivotal role in the pathogenesis of pulmonary fibrosis. TGF-β binds to type I and II serine/threonine receptor kinases on the cell surface, leading to the phosphorylation of SMAD2 and SMAD3. Subsequently, the phosphorylated receptor kinases are released into the cytoplasm where they form a complex with SMAD4 before translocating into the nucleus. Once inside, this activated Smad complex binds to specific Smad binding elements within the genome to execute its regulatory function, such as modulating fibrin expression (156, 157). Studies have shown that vitamin D can inhibit TGF-β-Smad signaling pathway (156, 158–160) through the specific process of 1,25(OH)_2_D_3_ binding complex with VDR, which directly interacts with SMAD3 to reduce the binding of SMAD3 to DNA, and ultimately inhibit TGF-β-Smad signal transduction (161, 162). In fact, it has been repeatedly demonstrated that 1,25(OH)_2_D_3_ interferes with the pro-fibrotic effects of TGF-β, which could in fact be predicted because calcitriol itself inhibits collagen synthesis in cells (163, 164). Moreover, vitamin D can also block the expression of several matrix metalloproteinases in alveolar macrophages and monocytes and participate in airway remodeling, which helps to reduce the excessive degradation and abnormal remodeling of the extracellular matrix, thus playing a protective role in the airways (165, 166). Additionally, vitamin D can reduce the proliferation of lung fibroblasts through the TGF-β pathway, thus playing a role in inhibiting pulmonary fibrosis (165, 167). Other studies have shown that vitamin D concentrations were positively associated with improvements in lung function, including ΔFEV1(%), ΔFVC(%), and ΔDLCO-SB(%) (166). Additionally, the presence of severe muscle weakness in individuals with IPF is associated with an elevated risk of mortality and disease severity due to cachexia or sarcopenia (168, 169). Previous studies have demonstrated the efficacy of vitamin D supplementation in enhancing muscle strength among patients afflicted with pulmonary disorders (170, 171).

The report of the Institute of Medicine (IOM) of the United States in 2011 not only discussed the upper limits (ULs) of the intake of high-dose vitamin D preparations during acute and short-term administration within a limited time period, but also emphasized the long-term administration of vitamin D. Acute toxicity may occur when the dose of vitamin D exceeds 10,000 international units per day, which will cause the serum 25(OH)D concentration exceeding 150 ng/ml (>375 nmol/L). This level is significantly higher than the upper limit of 4,000 international units per day recommended by the IOM. Taking a dose of more than 4,000 international units of vitamin D per day for several years may lead to the serum 25(OH)D concentration being in the range of 50 to 150 ng/ml (125 to 375 nmol/L), which may have potential chronic toxicity (172). Due to the high prevalence of vitamin D deficiency secondary to exocrine pancreatic insufficiency, it is a common practice to supplement vitamin D in the population of patients with cystic fibrosis. Thomas et al. conducted a retrospective analysis of the medical records of all cystic fibrosis patients followed up at the Cliniques universitaires Saint-Luc over the past 10 years. The results showed that in this high-risk population, the number of cases of excessive vitamin D intake and toxicity increased due to dosage errors in the preparation of magistral liposoluble vitamin preparations. The serum vitamin D levels of 5% of the patients indicated excessive vitamin D intake, and another 2 patients developed severe hypercalcemia (173).

### 4.5 Vitamin E

Vitamin E encompasses a group of antioxidant fat-soluble vitamins, including α-, β-, γ- and δ-tocopherol as well as α-, β-, γ- and δ-tocotrienols, of which α-tocopherol stands out as the most biologically active form of vitamin E (174). The role of vitamin E in pulmonary fibrosis seems to be controversial, however, certain reports have suggested its inhibitory effect on this condition. Similar to vitamin C, vitamin E also has potent antioxidant properties that directly inhibit ROS formation in radiation fibrosis patients (82). Dietary supplementation of vitamin E significantly reduced the extent of TGF-β1, collagen deposition and histological damage following intratracheal amiodarone administration (175), and vitamin E reduced amiodarone-induced cytotoxicity in pulmonary cells, whereas other antioxidant treatments were ineffective (176). Dietary vitamin E supplementation enables rapid vitamin accumulation in lung tissue and mitigates amiodarone-induced elevation of TGF-β and hydroxyproline levels, thereby preventing lung tissue damage. Another recent study demonstrated that vitamin E significantly attenuates bleomycin-induced pulmonary fibrosis by improving mitochondrial structure and function, modulating iron metabolism, reducing inflammation (significantly reducing the transcriptional levels of interleukin-6 (Il-6), interleukin-33 (Il-33), chemokine ligand 5 (Ccl5) and TNF-α, as well as inhibiting the fibrotic effects on epithelial cells and fibroblasts (177). However, Card et al. point out that although dietary supplementation also significantly increased lung mitochondrial vitamin E content, it did not ameliorate the mitochondrial respiratory depression and disruption of mitochondrial membrane potential caused by amiodarone (178).

The role of vitamin E in cystic fibrosis disease remains a subject of intense debate within current research. In theory, vitamin E is involved in protecting the airways from oxidative stress and inflammation. Previous studies have demonstrated a decline in serum α-tocopherol levels during lung deterioration, which subsequently returned to normal following intravenous antibiotic treatment (179–181). Even within the normal range, reduced serum levels of vitamin E are associated with an elevated incidence of lung deterioration in cystic fibrosis (179). It can be inferred that low serum α-tocopherol levels may be attributed to pulmonary inflammation rather than dietary vitamin E deficiency. Therefore, the degree of chronic pulmonary inflammation should be considered when studying the relationship between pulmonary fibrosis and serum α-tocopherol. However, in a single-walled carbon nanotube-induced pulmonary fibrosis animal experiment, the researchers found that dietary vitamin E deficiency increased lung inflammation and oxidative stress. After feeding mice a vitamin E-adequate and a vitamin E-deficient diet, the researchers found that the vitamin E-deficient mice showed a 90-fold decrease of α-tocopherol in lung tissue, and a significant decrease of antioxidants and increased inflammatory response (182). However, Woestenenk et al. found that serum α-tocopherol deficiency was also rare in a large sample of children and adolescents with cystic fibrosis, although the actual vitamin intake was lower than recommended, and the study did not find a protective effect of higher serum α-tocopherol on lung function in patients with cystic fibrosis (183). The study further notes that the recommended amount of vitamin E supplementation for cystic fibrosis patients in both Europe and North America is higher than needed to prevent deficiency, and may even lead to abnormal vitamin E levels (183). Supplementation of vitamin E exceeding 400 mg/day in patients with cystic fibrosis has demonstrated deleterious effects, including an elevated risk of mortality in adults (184) and occurrence of hemorrhagic stroke (185).

### 4.6 Vitamin K

Acute exacerbations of IPF are characterized by histopathological features of diffuse alveolar damage (DAD) or hemorrhage (DAH), and studies have found that vitamin K deficiency exacerbates DAH symptoms (186–188). In addition, another study showed approximately 29% of individuals under the age of 18 with cystic fibrosis exhibit diminished levels of vitamin K (189). The risk of clinically relevant vitamin K deficiency is heightened by various factors, such as the use of oral anticoagulants or antibiotics, as well as reduced intestinal vitamin K uptake for multiple reasons, such as fat uptake inhibitors (190). Janssen R. proposed that vitamin K deficiency is one of the potential sources of pathogenesis of IPF. This proposition builds on Booth and colleagues' finding that there is a significant increase in vitamin K-dependent matrix Gla protein (MGP) expression in the lungs of IPF patients, indicating potential vitamin K deficiency (191). Vitamin K plays an important role in preventing tissue degeneration caused by the hardening or degradation of elastin and collagen (192). The vitamin K-activated MGP possesses the capability to penetrate the interior of elastin and collagen fibers, thereby playing a pivotal role in safeguarding ECM proteins against mineralization (193). Growth arrest-specific 6 (GAS6) and protein S are also vitamin K-dependent proteins, which are important regulators of tissue repair after injury and may be involved in the pathogenesis of pulmonary fibrosis (194). In addition to anticoagulant effects, protein S also has a protective effect on lung fibers through anti-apoptotic properties (195). Therefore, assessing the vitamin K status of individuals with pulmonary fibrosis and providing tailored dietary recommendations to enhance their vitamin K levels could potentially serve as a valuable intervention strategy, particularly for those who frequently experience infections (requiring antibiotic treatment) while concurrently using anticoagulant medications.

Since the primary deficiency disease associated with vitamin K is bleeding due to impaired blood clotting, it is commonly believed that high intake of vitamin K may increase the risk of thrombosis. However, in fact, excessive intake of vitamin K does not lead to more carboxylation of clotting factors. Even when monitored with the most sensitive technique (endogenous thrombin potential, ETP), an increased tendency to thrombosis has not been found in any of the participants (196). At present, there is a lack of evidence on whether high intake of vitamin K has an adverse effect on patients with pulmonary fibrosis.

## 5 The potential value, limitations and future research directions of vitamins in the treatment of pulmonary fibrosis

Vitamins exhibit various advantages in the treatment of pulmonary fibrosis. Their targets of action include oxidative stress, inflammatory responses, and fibrosis-related signaling pathways. The possible targets of action for each vitamin are listed in Table 2. With low toxicity, high cost-effectiveness, and the potential to synergize with traditional therapies (such as pirfenidone), vitamins have become promising adjuvant therapeutic agents. However, several limitations impede the clinical translation of vitamins. Currently, the evidence mainly comes from preclinical models or small-scale studies, lacking sufficient data on long-term efficacy, optimal dosages, or inter-individual variations. There are also some ambiguities in the mechanisms of action. For example, folic acid has a dual effect on fibroblast proliferation and the alleviation of oxidative stress. Moreover, some seemingly contradictory effects further complicate the application of vitamins. High doses of vitamin C may act as a pro-oxidant, and excessive vitamin E can increase the risk of bleeding. Biomarkers for predicting treatment responses (such as serum 25-hydroxyvitamin D for detecting vitamin D levels) remain underdeveloped, which restricts the formulation of personalized treatment regimens. Future research must prioritize large-scale randomized trials to verify the clinical efficacy of vitamins and establish standardized treatment protocols. Combination treatment regimens are worthy of in-depth exploration. For instance, the combination of vitamins C and E can enhance the antioxidant effect, or the use of folic acid-conjugated drugs can achieve targeted drug delivery to macrophages, thereby improving the therapeutic effect. In addition, safety assessment is essential. It is necessary to clarify the upper limit of the safe dosage and address the issue of drug interactions. Filling these research gaps will help to fully unleash the potential of vitamins in the treatment of pulmonary fibrosis.

**Table 2:** The potential targets of various vitamins in pulmonary fibrosis.

**Vitamins**	**Potential targets**
Vitamin A	TGF-β/IL-6/IL-6R; PKC-δ/NF-κB; p38MAPK/NF-κB
Vitamin B	
Niacin	Calcium/PLA2; IκBα/NF-κB/(IL-1a, IL-6, TNF-α and TGF-β); iNOS
Folic acid	SLC25A32/MTHFD2; mitochondrial ROS
Vitamin C	Nrf2/Nox4; TGF-β1/Smad3; P53; caspase-3; Bax/Bcl-2; IL-17
Vitamin D	TNF-α/TF; TFPI; IL-3; IL-17; IL-1; IL-6; IL-8; TNF-α; IL-10; TGF-β/Smad
Vitamin E	TGF-β1; ROS; IL-6; IL-33; Ccl5; TNF-α; α-tocopherol
Vitamin K	MGP; GAS6; protein S;

## 6 Concluding remarks

New roles and uses for known substances are being discovered all the time, as are substances in our food. In the current era of escalating healthcare expenditures, cost-effective interventions hold great appeal. Numerous studies have demonstrated the significant role of vitamins in decelerating the progression of pulmonary fibrosis. Given the widespread psychological acceptance of vitamins as a relatively safe and economical natural compound, their utilization should be encouraged in regions with positive clinical data.
